# Determination of Lactose Concentration in Low-Lactose and Lactose-Free Milk, Milk Products, and Products Containing Dairy Ingredients, Enzymatic Method: Single-Laboratory Validation First Action Method 2020.08

**DOI:** 10.1093/jaoacint/qsab032

**Published:** 2021-03-16

**Authors:** Ruth Ivory, Elaine Delaney, David Mangan, Barry V McCleary

**Affiliations:** Megazyme, Bray Business Park, Southern Cross Rd, Bray A98 YV29, Ireland

## Abstract

**Background:**

The AOAC Stakeholder Panel on Strategic Food Analytical Methods issued a call for methods for the measurement of lactose in low-lactose and lactose-free products under *Standard Method Performance Requirement* (SMPR^®^) 2018.009. Megazyme’s Lactose Assay Kit (K-LOLAC) was developed specifically to address the need for accurate enzymatic testing in lactose-free samples.

**Objective:**

K-LOLAC was validated for measurement of lactose in low-lactose and lactose-free milk, milk products, and products containing dairy ingredients. A single-laboratory validation (SLV) of the method is reported.

**Method:**

K-LOLAC is an accurate and sensitive enzymatic method for the rapid measurement of lactose in low-lactose or lactose-free products. Validation analysis was performed on a sample set of 36 commercial food and beverage products and a set of 10 certified reference materials. Parameters examined during the validation included working range and linear range, selectivity, LOD, LOQ, trueness (*bias*), precision (repeatability and intermediate precision), robustness, and stability.

**Results:**

For all samples tested within the lower range (10–100 mg/100 g or mL), recoveries varied from 93.21–114.10%. Recoveries obtained for samples in the higher range (>100 mg/100 g or mL) varied from 94.44–108.28%. All materials had repeatability relative standard deviations (RSD_r_ and RSD_ir_) of <9%.

**Conclusions:**

The commercial K-LOLAC assay kit, as developed by Megazyme, meets the requirements set out under SMPR 2018.009.

**Highlights:**

K-LOLAC is a robust, quick, and easy method for analysis of lactose in foodstuffs and beverages.

Lactose is the most abundant sugar found in bovine milk, usually present at ∼5 g/100 mL (5% w/v). As a result, it is also typically found in a wide range of commercial dairy products including cheese, yoghurt, cream, butter, and whey. Following ingestion, lactose is normally hydrolyzed by lactase-phlorizin hydrolase (commonly referred to as lactase, β-galactosidase, EC. 3.2.1.108) in the human small intestine, with absorption of the released D-galactose and D-glucose (1). Humans lacking or deficient in this enzyme cannot digest lactose, which therefore passes undigested to the colon where it undergoes rapid microbial fermentation causing adverse gastrointestinal symptoms associated with lactose intolerance, such as nausea, cramps, bloating, and diarrhea (2).

Lactose intolerance or lactase non-persistence (LNP) affects approximately 65% of the global human population. The frequency of primary lactose intolerance varies greatly between ethnic and racial populations, with approximately 5% of northern European and greater than 90% of Southeast Asian populations being affected (3, 4).

To address the prevalence of lactose intolerance, dairy manufacturers have introduced low-lactose and lactose-free dairy products, which are typically manufactured by the use of a commercial β-galactosidase (EC 3.2.1.23) to hydrolyze the lactose present into glucose and galactose. While the US Food and Drug Administration (FDA) and the European Food Safety Authority (EFSA) have not issued overarching regulations on the allowable lactose thresholds in all products, a number of countries have defined “low-lactose” as containing less than 1 g lactose per 100 g product and “lactose-free” as containing less than 10–100 mg lactose per 100 g product. In EU legislation, the term “lactose-free” has been defined only for infant and follow-on formula as <10 mg per 100 kcal (5).

In 2018 the AOAC Stakeholder Panel on Strategic Food Analytical Methods issued a call for methods for the measurement of lactose in low-lactose and lactose-free products. The measurement of lactose has previously been described using a range of methods including IR spectroscopy (AOAC Method **972.16**) (6), polarimetry (AOAC Method **896.01**) (7), gravimetry (AOAC Method **930.28**) (8) along with various types of chromatographic and enzymatic methods. Traditional enzymatic methods have relied on the *β*-galactosidase-mediated hydrolysis of lactose and the subsequent measurement of the glucose or galactose released as a result (9, 10). These methods are well suited to the measurement of lactose in traditional dairy products in which lactose contents of ∼5 g/100 mL are usually found. However, due to the presence of galactosyl-glucosyl oligosaccharides in the lactose-free samples (some of which have the potential to interfere by inflating glucose released upon hydrolysis with β-galactosidase), the application of traditional enzymatic tests to the measurement of lactose in lactose-free products typically results in inaccurate quantitation, with overestimation of the residual lactose present being the most prevalent outcome (11). This necessitates the utilization of a β-galactosidase enzyme that is selective for lactose in the presence of these potentially interfering sugars.

Megazyme’s Lactose Assay Kit (K-LOLAC) was developed specifically to address the need for accurate enzymatic testing in lactose-free samples. It is an enzymatic method used for the rapid measurement of lactose in foodstuff and beverages. The Lactose Assay is simple, accurate, and sensitive, and suitable for the determination of lactose in low-lactose or lactose-free products, including infant formula and adult nutritional drinks, conventional dairy samples, and a variety of food samples. The method offers a sequential assay procedure for measurement of free D-glucose followed by lactose in the same reaction cuvette. Quantification is based on the hydrolytic activity of β-galactosidase, which hydrolyses lactose to glucose and galactose. Free D-glucose is first measured using a hexokinase (HK)/glucose 6-phosphate dehydrogenase (G-6PDH)/6-phosphogluconate dehydrogenase (6-PGDH) based assay procedure, and then β-galactosidase is added to hydrolyze the lactose in the same reaction vessel with concurrent measurement of the released D-glucose. The β-galactosidase employed is selective for lactose and the determined lactose values are similar to those obtained by ion chromatography (12). The free glucose measurement is complete within 10 min and the subsequent lactose measurement is complete within 15 min. The method includes pre-treatment steps to clarify and deproteinate samples and also to remove the high levels of free D-glucose in the samples, allowing accurate measurement of lactose at very low levels. This method measures lactose in mg/100 mL or g/100 mL (liquid samples) and mg/100 g or g/100 g (solid samples).

##  

### *AOAC* Official Method^SM^*2020.08* Lactose Concentration in Low-Lactose and Lactose-Free Milk, Milk Products, and Products Containing Dairy     Ingredients High Sensitivity Enzymatic Nethod       (Megazyme Test Kit K-LOLAC)           First Action 2020

[Applicable for the determination of lactose concentration in a variety of lactose-free and low lactose samples including milk, milk products, infant formula, adult nutritional products, and products containing dairy ingredients.]

*Caution*: The general safety measures that apply to all chemical substances should be adhered to. For more information regarding the safe usage and handling of the reagents and components please refer to the associated Material Safety Data Sheet (MSDS) that is available from the manufacturer’s website (www.megazyme.com).

This test should not be carried out by anyone other than a trained and experienced laboratory analyst. A lactose control should be included with analysis if there is any doubt as to the performance of the reagents or the performance of the analyst.

### A. Principle

This enzymatic method relies on the β-galactosidase mediated hydrolysis of lactose and the subsequent measurement of the glucose released. This method describes the selective hydrolysis of lactose by MZ104 β-galactosidase, with further selectivity achieved by use of a linear extrapolation calculation to remove the contribution from interfering sugars commonly found in lactose-free samples, such as β-1,6-lactose (allolactose), that are slowly hydrolyzed during the incubation.

Free D-glucose is efficiently removed from the sample by conversion to D-gluconic acid using the glucose oxidase/catalase pre-treatment system. Prior to lactose hydrolysis, any remaining free D-glucose is phosphorylated by the enzyme HK, in the presence of adenosine-5’-triphosphate (ATP) to glucose-6-phosphate (G-6-P) with the simultaneous formation of adenosine-5’-diphosphate (ADP). G-6-P is oxidized by the enzyme glucose-6-phosphate dehydrogenase (G6P-DH) in the presence of nicotinamide-adenine dinucleotide phosphate (NADP^+^) to gluconate-6-phosphate (gluconate-6-P) with the formation of reduced nicotinamide-adenine dinucleotide phosphate (NADPH). Gluconate-6-P is immediately converted to D-ribulose-5-phosphate (R-5-P), carbon dioxide (CO_2_), and a further molecule of NADPH by the enzyme 6-phosphogluconate dehydrogenase (6-PGDH). Lactose is then hydrolyzed to D-galactose and D-glucose by β-galactosidase and D-glucose released enters the series of reactions catalyzed by HK, G6P-DH, and 6-PGDH. The amount of NADPH formed is stoichiometric to twice the amount of lactose as two molecules of NADPH are produced for each D-glucose molecule originating from the lactose in the sample.

### B. Equipment

- *Volumetric flasks and glass beakers.—*50 and 100 mL.
- *Disposable plastic microfuge tubes.—*2 mL.
- *Disposable polypropylene tubes.—*13 mL.
- *Disposable plastic cuvettes.—*1 cm light path, 1.5 mL.
- *Micro-pipettors.—*e.g., Gilson Pipetman^®^ P20 and P100.
- *Analytical balance*.
- *Magnetic stir plate.—*With heating capability (required max temperature 70°C).
- *Boiling water bath.—*Required temperature 100°C.
- *Microfuge.—*Required speed 13 000 rpm.
- *UV-VIS spectrophotometer.—*Required wavelength 340 nm.
- *Vortex mixer.*
- *Filter papers.—*e.g., Whatman No. 1, 9 cm.

### C. Chemicals and Reagents

Reagents **a–g** are supplied in the Megazyme Lactose Assay Kit (K-LOLAC).

- *Solution 1*.—Bottle 1 containing buffer (pH 8.0), MgCl_2_ plus sodium azide (0.02% w/v) as a preservative. Use the contents of Bottle 1 as supplied. Stable for >3 years at 4°C.
- *Solution 2*.—Bottle 2 containing glucose oxidase and catalase, lyophilized powder. Stable for >3 years below –10°C. For use, dissolve the contents of Bottle 2 in 14 mL of distilled water. To avoid repetitive freeze/thaw cycles, divide into appropriately sized aliquots and store in polypropylene tubes below –10°C. Stable for >3 years below –10°C.
- *Solution 3*.—Bottle 3 containing buffer (pH 7.6), MgCl_2_, KCl plus sodium azide (0.02% w/v) as a preservative. Use the contents of Bottle 3 as supplied. Stable for >3 years at 4°C.
- *Solution 4*.—Bottle 4 containing NADP^+^, ATP, PVP, mannitol plus sodium azide as a preservative. Stable for >3 years below –10°C. For use, dissolve the contents of Bottle 4 in 4 mL of distilled water. To avoid repetitive freeze/thaw cycles, divide into appropriately sized aliquots and store in polypropylene tubes. Stable for >3 years below –10°C.
- *Suspension 5*.—Bottle 5 containing HK glucose 6-phosphate dehydrogenase, 6-phosphogluconate dehydrogenase plus sodium azide (0.02% w/v) as a preservative. Use the contents of Bottle 5 as supplied. Before opening for the first time, shake the bottle to remove any enzyme that may have settled on the stopper. Subsequently store the bottle in an upright position. Swirl the bottle to mix contents before use. Stable for >3 years at 4°C.
- *Suspension 6*.—Bottle 6 containing MZ104 β-galactosidase suspension plus sodium azide (0.02% w/v) as a preservative. Use the contents of Bottle 6 as supplied. Before opening for the first time, shake the bottle to remove any enzyme that may have settled on the stopper. Subsequently store the bottle in an upright position. Swirl the bottle to mix contents before use. Stable for >3 years at 4°C.
- *Solution 7*.—Bottle 7 containing lactose standard solution (25 mg/100 mL) plus sodium azide (0.02% w/v) as a preservative. Use the contents of Bottle 7 as supplied. Stable for >3 years at 4°C
  Reagents **h–j** are additional reagents and not supplied in the Lactose Assay Kit.
- *Concentrated Carrez I solution*.—200 mL. Dissolve 30 g of potassium hexacyanoferrate (II) trihydrate (K_4_[Fe(CN)_6_]·3H_2_O) in 200 mL of distilled water. Stable for >3 years at room temperature.
- *Concentrated Carrez II solution*.—200 mL. Dissolve 60 g of zinc sulphate heptahydrate (ZnSO_4_·7H_2_O) in 200 mL of distilled water. Stable for >3 years room temperature.
- *Hydrogen peroxide (H_2_O_2_, ∼30% w/w).*—Use as purchased. Refer to manufacturer guidelines for safety and stability information.

### D. Sample Preparation

- *Sample extraction procedure*.—“Ready-to-feed samples (infant formula, whey protein, adult nutritional drinks, and other samples intended to be consumed in beverage form). *Note*: Solid samples that are intended to be consumed in liquid form should be prepared in liquid form before testing using the **D(b)** . A sample preparation example is outlined below.
  1. Accurately weigh 6.25 g of powdered sample into a 50 mL glass beaker. Record the weight.
  2. Add a stir bar and ∼30 mL of distilled water at ∼40°C.
  3. Mix on a magnetic stir plate for ∼15 min.
  4. Quantitatively transfer to a 50 mL volumetric flask.
  5. Dilute to volume (50 mL) with distilled water.
  6. Take 0.5 mL of liquid sample for **D(b)**.
- *Sample extraction/clarification procedure.—*Liquid samples*.*
  1. Ensure that milk sample has been mixed thoroughly before sampling.
  2. Pipette the following reagents into a 2 mL disposable microfuge tube:
    0.9 mL of distilled water (at room temperature, ∼20–25°C).
    0.5 mL of milk sample.
    0.05 mL of Carrez II solution.
    0.05 mL of Carrez I solution.
    Cap the tube and mix by vortex.
  3. Centrifuge at 13 000 rpm for 10 min.
  4. Take 1.0 mL of the clear filtrate for **D(e)**.
- *Sample extraction/clarification procedure.—*Solid samples.
  1. Accurately weigh ∼10 g of solid sample into a 50 mL glass beaker.
  2. Add a stir bar and ∼30 mL of distilled water.
  3. Mix on a magnetic stir plate and heat until temperature reaches 50°C. Continue stirring at temperature for ∼15 min or until sample has solubilized or homogenized.
  4. Quantitatively transfer to a 50 mL volumetric flask.
  5. Add 0.5 mL of Carrez II solution and mix.
  6. Add 0.5 mL of Carrez I solution and mix.
  7. Dilute to volume (50 mL) with distilled water.
  8. Filter an aliquot, discarding the first few mL of filtrate (∼5 mL).
  9. Take 1.0 mL of the clear filtrate for **D(e)**
- *Additional sample preparation example.—*Cheese solubilization*.* The melting point of cheese can vary based on parameters such as moisture content and age. A solubility assessment should be carried out by visual inspection. Although the solution may not be free of turbidity after solubilization, there should be no lumps present or noticeable lack of homogeneity. A sample preparation example is outlined below.
  1. Using a standard kitchen hand grater, grate cheese through a fine sieve.
  2. Accurately weigh ∼10 g of grated sample into a 50 mL glass beaker.
  3. Add a stir bar and ∼30 mL of distilled water.
  4. Mix on a magnetic stir plate and heat until temperature reaches 50°C. Continue stirring at temperature for ∼15 min and assess the level of solubilization.
  5. If the cheese has not solubilized at 50°C slowly increase the temperature by 5°C and further assess the level of solubilization.
  6. Continue to increase the temperature in increments of 5°C up to a temperature of 70°C if solubilization or homogenization has not occurred at lower temperatures.
  7. Quantitatively transfer to a 50 mL volumetric flask.
  8. Add 0.5 mL of Carrez II solution and mix.
  9. Add 0.5 mL of Carrez I solution and mix.
  10. Dilute to volume (50 mL) with distilled water.
  11. Filter an aliquot, discarding the first few mL of filtrate (∼5 mL).
  12. Take 1.0 mL of the clear filtrate for **D(e)**.
- *Glucose oxidase/catalase pre-treatment procedure.—All samples.*
  1. Pipette the following reagents to a 13 mL polypropylene tube:
    0.4 mL of distilled water (at room temperature, ∼20–25°C).
    1.0 mL of clear supernatant.
    0.1 mL of Solution 1.
    0.2 mL of Solution 2.
    0.1 mL of Hydrogen peroxide (H_2_O_2_, ∼30% w/w).
    Cap the tube and mix by vortex.
  2. Incubate at room temperature (20–25°C) for 15 min.
  3. Slowly loosen the cap to release pressure and then re-tighten.
  4. Incubate in a boiling water bath (100°C) for 5 min.
  5. Remove from water bath and allow to cool for ∼5 min.
  6. Transfer ∼1.0 mL of the solution to a 2 mL microfuge tube and centrifuge at 13 000 rpm for 10 min.
  7. Carefully pipette the required volume (0.1–0.4 mL) for use in **D(f)**. *Note:* The volume pipetted depends on the sample type. For lactose-free samples, the maximum volume of 0.4 mL should be transferred. For low lactose samples, a volume of 0.1 mL should be transferred.
- *Enzymatic determination reaction.—All samples.*
*Note:* For sample cuvettes, the amount of distilled water added should be adjusted depending on the sample volume required (i.e., 0.9 mL of distilled water where 0.1 mL of sample is used and 0.6 mL of distilled water where 0.4 mL of sample is used).
  1. Set the spectrophotometer to read absorbance at 340 nm.
  2. Blank the spectrophotometer against air or water.
  3. Prepare a blank cuvette by addition of the following reagents to a 1.5 mL UV cuvette:
    1.0 mL of distilled water.
    0.1 mL of Solution 3.
    0.05 mL of Solution 4.
    Cap the cuvette using a cuvette cap or parafilm and mix by gentle inversion.
  4. Prepare sample cuvettes (in duplicate) by addition of the following reagents to a 1.5 mL UV cuvette:
    0.9 mL or 0.6 mL of distilled water.
    0.1 mL or 0.4 mL of sample solution [**D(e)**].
    0.1 mL of Solution 3.
    0.05 mL of Solution 4.
    Re-cap the cuvette using the matching cuvette cap or parafilm and mix by gentle inversion.
  5. If required prepare a lactose standard cuvette by addition of the following reagents to a 1.5 mL UV cuvette:
    0.9 mL of distilled water.
    0.1 mL of Solution 7.
    0.1 mL of Solution 3.
    0.05 mL of Solution 4.
    Re-cap the cuvette using the matching cuvette cap or parafilm and mix by gentle inversion.
  6. After 3 min at 25°C read the absorbances of the blank and then samples (A_1_).
  7. Remove the cuvette caps or parafilm, taking care to avoid spillage of liquid.
  8. Start the reaction by addition of 0.02 mL of Suspension 5. Re-cap the cuvettes with the matching cap or parafilm after addition and mix by gentle inversion.
  9. After 10 min at 25°C read the absorbances of the blank and samples (A_2_).
  10. Remove the cuvette caps or parafilm, taking care to avoid spillage of liquid.
  11. Start the next reaction by addition of 0.02 mL of Suspension 6. Re-cap the cuvettes with the matching cap or parafilm after addition and mix by gentle inversion.
  12. After 15 min at 25°C read the absorbances of the blank and samples (A_3_).
  13. After a further 5 min (20 min from addition of Suspension 6) at 25°C read the absorbances of the blank and samples (A_4_).
  14. After a further 5 min (25 min from addition of Suspension 6) at 25°C read the absorbances of the blank and samples (A_5_).

### E. Calculations

Determine the value for A_3_ “creep corrected” (A_3cc_) using the MegaCalc software available on the manufacturer’s website, or the equation of the line calculation as outlined below.

A_3cc_ can be calculated as follows:

First, calculate the slope of the line (*m*).

$$m=\;\frac{y2\;-y1}{x2\;-\;x1}$$

where *y*2 = absorbance value A5 (measured after 25 min); *y*1 = absorbance value A3 (measured after 15 min); *×*2 = time of measurement for A5 reading (25); *×*1 = time of measurement for A3 reading (15).

Then, calculate the *y* intercept A_3cc_

$$A_{3\text{cc}}=y\;–\;\text{mx}$$

where *y* = absorbance at A3; *x* = time of measurement A3 (15).

Use the value for A_3cc_ in the calculation of ΔA_lactose_ below.

Determine the absorbance difference caused by hydrolysis of lactose (A_3cc—_A_2_) for both blank and sample. Subtract the absorbance difference of the blank from the absorbance difference of the sample, thereby obtaining ΔA_lactose_. The value of ΔA_lactose_ should as a rule be in the range of 0.02–1.2 absorbance units to achieve sufficiently accurate results.

The concentration of lactose can be calculated as follows:

$$c_{\mathrm{lactose}}=\;\frac{V\;\times \;\mathrm{MW}}{\varepsilon \times d\times v\times 2\;}\;\times \;{\Delta A}_{\mathrm{lactose}}\times \;100\;\times \;F\;[\mathrm{mg}/100mL]$$

where V = enzymatic determination reaction final volume [mL] = 1.19; MW = molecular weight of lactose [g/mol] = 342.3; ε = extinction coefficient of NADPH at 340 nm [l × mol^−1^ × cm^−1^] = 6300; *d* = light path [cm] = 1; *v* = sample volume [mL] = 0.1 or 0.4; 2 = 2 moles of NADPH produced for each mole of D-glucose or lactose; 100 = conversion to mg/100 mL; *F* = dilution factor.

For liquid and “ready to feed” samples

$$\frac{V1}{v1}\;*\;\frac{V2}{v2}=\frac{1.5}{0.5}\;*\;\frac{1.8}{1.0}=5.4$$

where *V*1 = final volume in liquid extraction procedure [mL]; *v*1 = sample volume in liquid extraction procedure [mL]; *V*2 = final volume in glucose oxidase/catalase treatment [mL]; *v*2 = sample volume in glucose oxidase/catalase treatment [mL].

### For solid samples


$$\frac{V2}{v2}=\frac{1.8}{1.0}=1.8$$

where *V*2 = final volume in glucose oxidase/catalase treatment [mL]; *v*2 = sample volume in glucose oxidase/catalase treatment [mL].

It follows for solid samples or “ready to feed” samples:

$$\left[\text{mg}/100g\right]=\frac{c_{\text{lactose}}\left[\text{mg}/\text{mL}\right]}{\text{extract}\;\text{concentration}\;\left[g/\text{mL}\right]}\times 100$$

where extract concentration = 0.2.

$$\text{Solid}\;\text{samples}=\frac{10g}{50\;\text{mL}}$$

or extract concentration = 0.125.

$$\text{Ready}\;\text{-to-}\;\text{Feed}=\frac{6.25g}{50\;\text{mL}}$$

where 100 = conversion to mg/100g.

## Results 

### Planning

**Figure 1.**
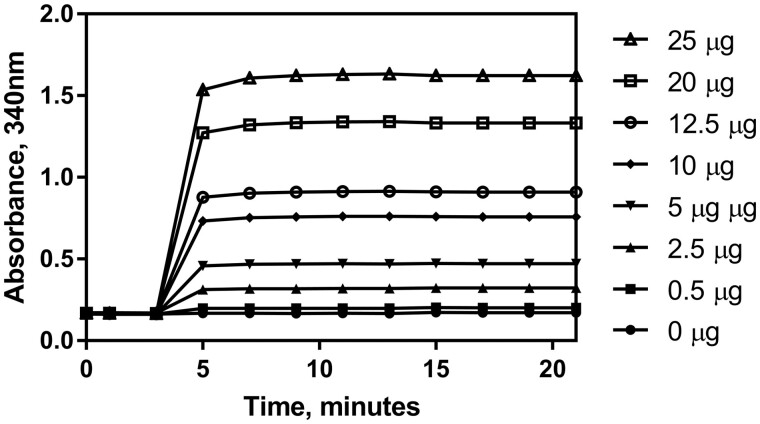
Reaction kinetic graph showing the time of reaction completion for glucose after addition of HK/G6PDH/6PGDH using a range of glucose concentrations across the linear range of the assay (0–25 μg glucose per test).

**Figure 2.**
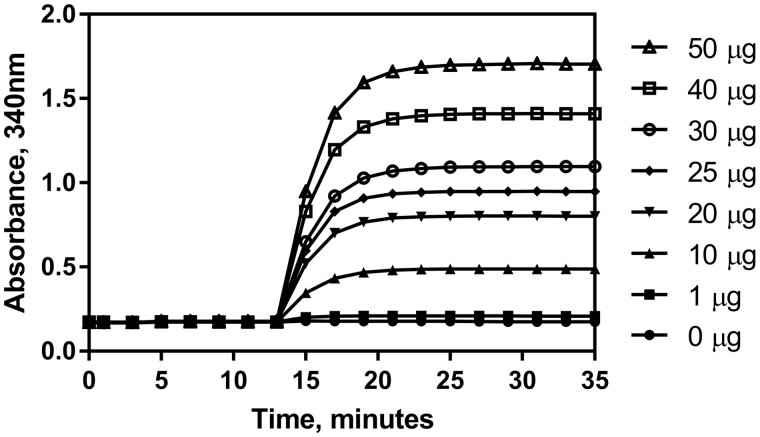
Reaction kinetic graph showing the time of reaction completion for lactose after addition of β-galactosidase using a range of lactose concentrations across the linear range of the assay (0–50 μg lactose per test). HK/G6PDH/6PGDH suspension was added after 3 min and β-galactosidase suspension was added after 13 min.

The purpose of these experiments was to verify and validate the Lactose Assay Kit (K-LOLAC) for the analysis of lactose concentration in low-lactose and lactose-free milk, milk products, and products containing dairy ingredients. The assay requires the addition of two enzymes to begin the reactions. Absorbance (A_2_) was taken 10 min after the addition of the HK glucose-6-phosphate dehydrogenase, 6-phosphogluconate dehydrogenase suspension (Suspension 5, hereafter referred to as HK/G6PDH/6PGDH). Absorbances (A_3_, A_4_ and A_5_) were taken 15, 20, and 25 min after the addition of the β-galactosidase (Suspension 6). All absorbances were read at 340 nm and the enzymatic determination reactions were carried out at 25°C unless stated otherwise. Samples that exhibited a “creep” in the reaction curve as outlined in the method section were corrected by extrapolation calculation. The creep reaction is described in more detail under *Selectivity*. The first reaction is complete within approximately 5 min, as shown in Figure 1, and the second reaction is complete within approximately 12.5 min, as shown in in Figure 2. Nonetheless, it is recommended that the user measure absorbance 10 min and 15, 20, and 25 min after enzyme addition for A_2_, A_3_, A_4_, and A_5_ respectively.

### Performance Characteristics

Performance characteristics that were investigated included working range, LOD, LOQ, trueness (*bias*), precision (reproducibility and repeatability), interference, selectivity, robustness, and stability.

#### (a) Working range.—

**Figure 3.**
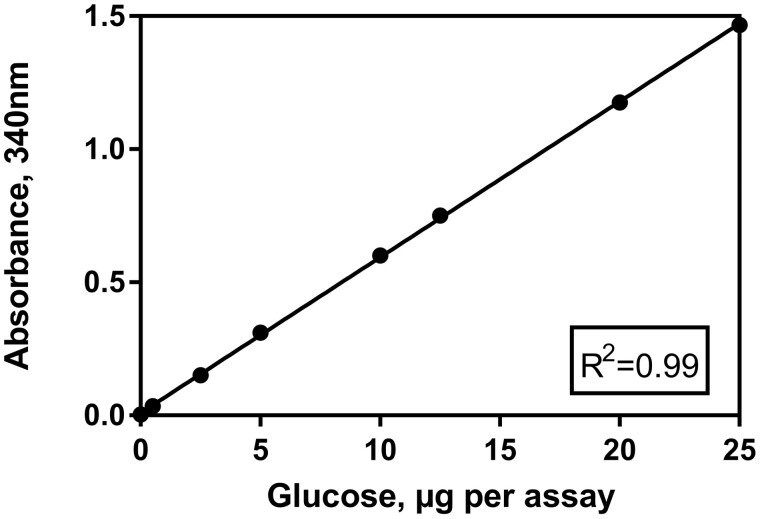
Examination of the linearity of the assay over a range of glucose concentrations (0–25 μg glucose per test).

**Figure 4.**
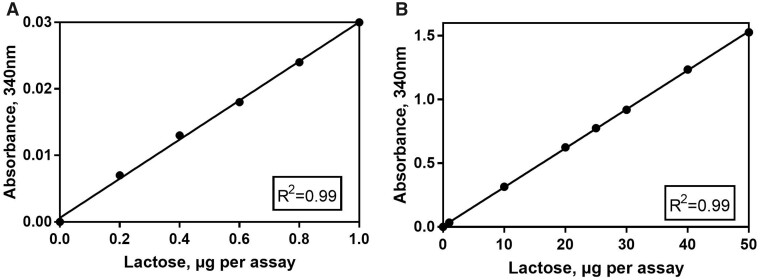
Examination of the linearity of the assay over a range of lactose concentrations; (A) 0–1 μg lactose per test and (B) 0–50 μg lactose per test.

For glucose (A_2_), 0.1 mL of a glucose standard was used as sample across a range of concentrations (1–25 mg/100 mL) corresponding to 1–25 µg of glucose per cuvette (Figure 3). For lactose (A_3_), 0.1 mL of lactose standard was used as sample across a range of concentrations (0.2–50 mg/100 mL lactose) corresponding to 0.2–50 μg of lactose per cuvette (Figure 4).

For liquid samples treated as per **D(b)** the working range of 1–50 µg of lactose per cuvette equates to a concentration range of 1.35–67.5 mg/100 mL in original sample when using the maximum volume of 0.4 mL in **D(f)**.

For solid or semi-solid samples treated as per **D(c)** the working range of 1–50 µg of lactose per cuvette equates to a concentration range of 2.25–112.5 mg/100g in original sample when using the maximum volume of 0.4 mL in the enzymatic determination reaction.

**Figure 5.**
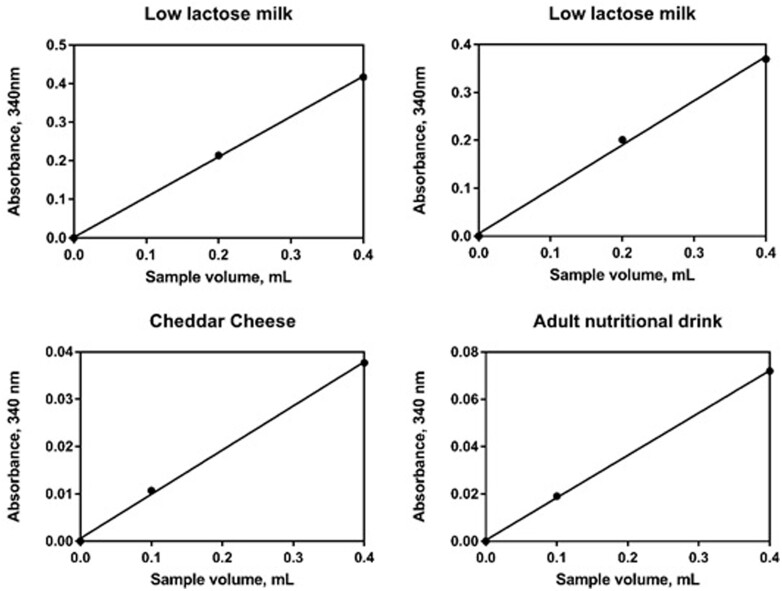
Examination of the linearity of the assay when increasing sample size across a range of real samples.

Linearity was also examined for a variety of real samples containing a range of lactose concentrations (Figure 5).

#### (b) LOD and LOQ.—

The LOD is the lowest concentration of the analyte that can be detected by the method. LOD is calculated as 3 × s0; where s0 is the standard deviation of a number of blank A_3_–A_2_ readings. The LOQ is the lowest level at which the assay performance is acceptably repeatable. LOQ is calculated as kQ × s0; where s0 is the standard deviation of a number of blank A_3_–A_2_ reading. The International Union of Pure and Applied Chemistry (IUPAC) default value for kQ is 10.

For samples containing lactose, with no pre-treatment, treated as per **D(f)** and using the maximum sample volume of 0.4 mL, the LOD is 0.05 mg/100 mL and the LOQ is 0.16 mg/100 mL (s0 = 0.002, *n *=* *20).

The above detection limits are for samples as used in the assay (i.e., after any required sample preparation such as glucose removal and deproteinization). The dilution used in pre-treatment must be accounted for while establishing the detection limits for specific samples. For liquid or milk samples treated as per **D(b)** and using the maximum sample volume of 0.4 mL the LOD is 0.27 mg/100 mL and the LOQ is 0.89 mg/100 mL (s0 = 0.002, *n *=* *20). For solid or semi-solid samples treated as per **D(c)** and using the maximum sample volume of 0.4 mL the LOD is 0.44 mg/100 g and the LOQ is 1.47 mg/100 g (s0 = 0.002, *n *=* *20). Where sample analysis returned an absorbance value lower than the absorbance value as determined for the LOQ calculation (i.e., an absorbance value lower than 0.03), these samples were reported as containing “less than” the LOQ values specified (i.e., <0.89 mg/100 mL for liquid samples and <1.47 mg/100 g for solid samples).

#### (c) Trueness (bias).—

Comparison of the mean of the results (x) achieved with the Lactose Assay Kit *Enzymatic determination reaction.—All samples* procedure with a suitable reference value (x_ref_) (Table 1). Relative bias is calculated in percent as:

$$b\left(\%\right)=x\;–{\;x}_{\text{ref}}/x_{\text{ref}}x\;100$$

**Table 1.** Relative bias [*b*(%)] using a single lactose standard, the enzymatic determination reaction, using 0.1 mL of standard per test

	*n*	Ref material, mg/100mL	Mean, mg/100mL	*b*(%)
Lactose	30	25	25.06	0.24

**Table 2.** Relative bias [*b*(%)] using a variety of internal lactose standards in the enzymatic determination reaction after sample treatment as per **D(b)** and the **D(e)** using 0.1 mL of sample in the enzymatic determination reaction

	*n*	Lactose in cuvette, µg	Ref material, mg/100 mL	Mean, mg/100 mL	*b*(%)
Lactose	8	50	270.0	272.5	0.94
	8	40	216.0	216.6	0.29
	8	20	108.0	109.0	1.01
	8	10	54.0	55.4	2.67
	8	2	10.8	10.9	1.19
	8	1	5.4	5.5	2.10
	8	0.5	2.7	2.6	−2.69

The reference material for this purpose is lactose supplied with the Lactose Assay Kit (K-LOLAC) at 25 mg/100 mL.

Comparison of the mean of the results (x) achieved with the Lactose Assay Kit **D(b)** procedure with a variety of suitable reference values (x_ref_) across the liner range of the assay (Table 2). Relative bias is calculated in percentage as:

$$b\left(\%\right)=x\;–{\;x}_{\text{ref}}/x_{\text{ref}}x\;100$$

The reference materials for this purpose are lactose standards formulated in-house from 2.7–270 mg/100 mL.

Trueness was also assessed using MUVA-Kempten certified reference materials. Reference values (x_ref_) were provided by the manufacturer based on results achieved by HPLC. Relative bias is calculated in percentage as:

$$b\left(\%\right)=x\;–{\;x}_{\text{ref}}/x_{\text{ref}}x\;100$$

Results are reported in Table 3. A poor recovery was observed for sodium caseinate (MUVA-CA-0904) and was initially attributed to what appeared to be the poor solubility of this material in water using the standard extraction procedure. Further studies were undertaken and solubility appeared to improve, however the lactose recovery did not improve. Examination of the specification sheet supplied with the material showed that the manufacturer specifies a standard deviation of 0.01 g/100g for this material. Given the specified value (x_ref_) of 0.026 g/100g and the measured value (x) of 0.021 g/100g (a value within the specified deviation range), statistical analysis was carried out with the null hypothesis that the measured value should not be considered significantly different than the value specified. A two-tailed two-sample *t*-test was conducted. There was no significant difference between the mean for expected result (M = 0.026; SD = 0.01) and the mean for the measured result (M = 0.021; SD = 0.00065); *t *=* *1.558 (df 14), *P* = 0.05.

**Table 3.** Relative bias [*b*(%)] using a number of certified reference materials, after sample extraction and pre-treatment as appropriate for each sample matrix and analysis in the enzymatic determination reaction

Sample	Expected lactose, g/100g (xref)	Measured lactose, g/100g	Recovery, %	*b*(%)
CRM1	0.007	0.006	90.09	−11.00
CRM2	0.212	0.205	96.74	−3.37
CRM3	0.026	0.021	80.76	−19.23
CRM4	4.917	4.995	101.59	0.14
CRM5	24.277	23.954	98.67	−1.35
CRM6	22.100	21.397	96.82	−3.28
CRM7	22.670	22.872	100.89	0.88
CRM8	2.810	2.638	93.89	−6.50
CRM9	4.060	3.794	93.46	−7.00
CRM10	9.440	8.737	92.56	−8.04

#### (d) Precision.—

Assay repeatability (RSD_r_) using the **D(f)** procedure was determined using a series of standards (formulated in-house and ranging from 2.7–270 mg/100 mL; Table 4). These materials were analyzed in order to provide system repeatability data (i.e., the suitability of the system for analysis of the specified lactose concentrations, disregarding any possible matrix or sample influence on repeatability).

Sample repeatability (RSD_r_) and sample intermediate precision (RSD_ir_) was determined by analysis of seven commercial samples (infant formulas, lactose free milks, and cheeses) with lactose concentrations between 2.1–135 mg/100 mL, in order to provide sample repeatability data (i.e., the suitability of the system for analysis of the specified sample type). For RSD_r_ (repeatability) the experiments were carried out by one analyst over a period of 3 days, with two extractions per sample per day, analyzed in duplicate. For RSD_ir_ (intermediate precision), these results were compared to results achieved by a second analyst using separate reagents and equipment over a period of 3 days, also with two extractions per sample per day, analyzed in duplicate. Each “extraction” as described in the table refers to a separate dilution of the sample material by the analyst (Table 5).

#### (e) Recovery.—

Recovery was first assessed by addition of a known amount of lactose to the extraction, without sample, for both the solid and liquid sample extraction and clarification procedures (i.e., the recovery of a lactose spike, disregarding any possible matrix or sample influence on recovery).

Real samples were then tested, by spiking these samples with known quantities of lactose in the initial extraction phases, prior to treatment for assay. The recoveries were assessed by comparison to sample assays (without spike) and assay of lactose spike (without sample). A wide range of samples was chosen for recovery testing in order to obtain as much information and cover as many sample matrixes as possible. Table 6 contains recovery data, showing the percentage recovery of lactose spike for the 36 samples. Spiking of real samples (including lactose-free milks, infant formulas, adult nutritional drinks, yoghurts, food samples, cheeses) resulted in recoveries between 93 and 114%.

**Table:** Table 4**.** Repeatability (RSD_r_) using a range of internal lactose standards in the enzymatic determination reaction after sample treatment as per **D(b)** and **D(e)**

Analyte	*n*	Lactose in cuvette, µg	Ref material, mg/100 mL	Mean, mg/100 mL	STDEV^a^	%CV^b^
Lactose	8	50	270.0	272.5	3.57	1.31
	8	40	216.0	216.6	2.32	1.07
	8	20	108.0	109.0	0.93	0.86
	8	10	54.0	55.4	1.03	1.85
	8	2	10.8	10.9	0.31	2.78
	8	1	5.4	5.5	0.17	3.11
	8	0.5	2.7	2.6	0.16	6.22

^a^STDEV = Standard Deviation.  ^b^%CV = Coefficient of variation (%).

#### (f) Robustness.—

Occasionally the user may lack the capability to control certain factors during the test (e.g., temperature at which the assay is run or time at which the absorbance is measured). Robustness experiments were carried out to show that the method does not suffer due to minor variations or adjustments to certain parameters.

**Figure 6.**
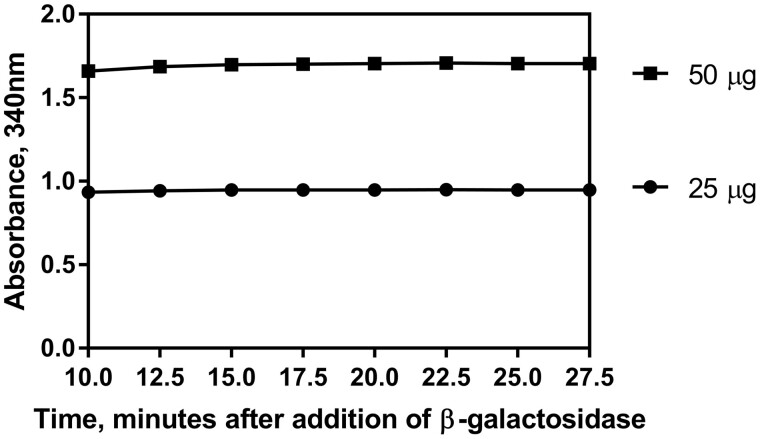
Examination of the robustness of the assay by adjusting the time at which the absorbance measurement for lactose is taken using two concentrations of lactose at the middle and the top of the linear range (25 and 50 μg). Absorbance at 340 nm was measured at 10, 12.5, 15 (recommended for assay), 17.5, 22.5, 25, and 27.5 min after addition of β-galactosidase.

*(1) Time of measurement*.—The time at which absorbance measurement (A_3_) is taken was also evaluated for robustness. In general, the enzymatic determination reaction is complete within 15 min of addition of β-galactosidase (Suspension 6). The assay was carried out as per the procedure (at 25°C) using the Megazyme lactose standard solution (Solution 7). Figure 6 shows that the results do not suffer when adjustments are made to the time at which the absorbance measurement is made. Results remain the same when absorbance values are taken at 10, 12.5, 15 (recommended for assay), 17.5, 22.5, 25, and 27.5 min.

**Figure 7.**
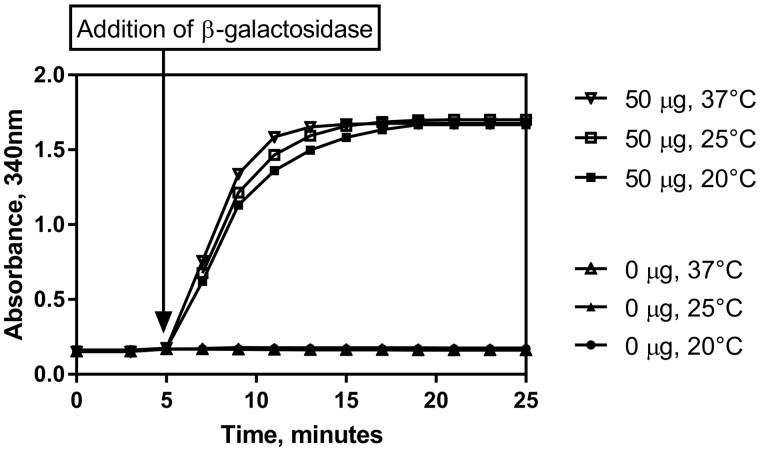
Examination of the robustness of the assay by adjusting the temperature at which the enzymatic determination reaction takes place. Assay carried out at 20, 25, and 37°C with 0 and 50 μg of lactose per test. The β-galactosidase suspension was added after 5 min.

*(2) Assay temperature*.—The temperature at which the enzymatic determination reaction takes place was examined as part of the robustness experiments. The assay was carried out as per the **D(f)** procedure using the lactose standard solution (Solution 7) at the top of the linear range (50 µg per test) over a range of temperatures. Figure 7 shows that the results do not suffer when adjustments are made to the temperature at which the assay is carried out. Results remain the same after incubation at 20, 25, and 37°C though there is a small variation in the time to completion of the assay. This variation in time to completion does not negatively affect the result of the assay as absorbance (A_3_) should be read at 15 min.

**Figure 8.**
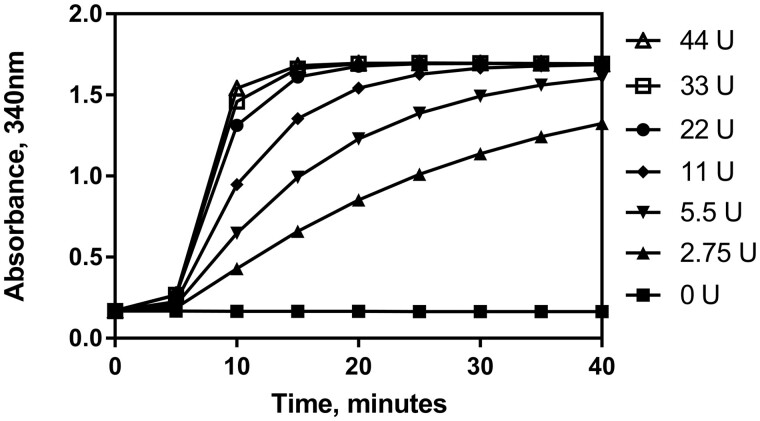
Examination of the robustness of the assay by adjusting the concentration of β-galactosidase enzyme in the enzymatic determination reaction. Assay carried out with 50 μg of lactose per test. β-galactosidase suspension (0.02 mL, 137.5 U/mL to 2200 U/mL) was added after 5 min.

*(3) Units of β-galactosidase added*.—Dilutions of the β-galactosidase suspension (Suspension 6) were made in 3.2 M ammonium sulphate in order to create a range of enzyme concentrations (137.5–2200 U/mL, the equivalent of 2.75–44 units per test when using 0.02 mL). 0.02 mL of each suspension was added to the test as in the **D(f)** procedure. The assay was carried out using the lactose standard solution (Solution 7) at the top of the linear range (50 µg per test) and results can be seen in Figure 8. A reduction in the number of units of β-galactosidase per test caused a reduction in time-to-completion of assay, however it can be seen that the β-galactosidase as supplied (2500 U/mL, the equivalent of 50 units per test when using 0.02 mL) is greatly in excess of the 22 units per test required to complete the reaction within the time frame specified for measurement in the enzymatic determination reaction.

**Figure 9.**
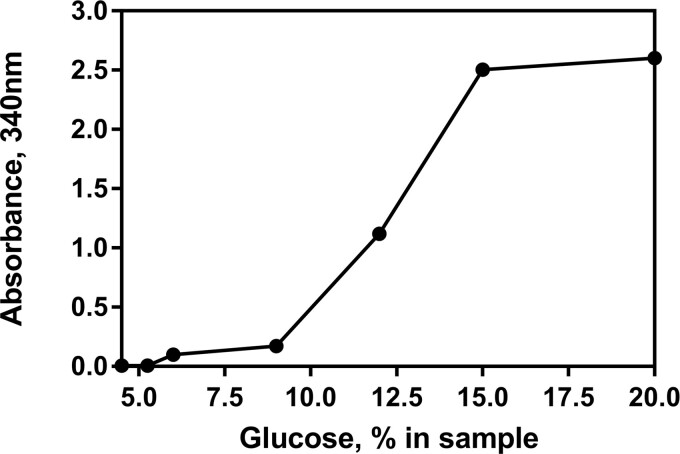
Examination of the robustness of the sample pre-treatment step by extraction of a range of glucose solutions as per **D(e)** and analysis using **D(f)**. Measured absorbance for glucose (A2) is shown on the *y* axis.

*(4) Glucose removal*.—The robustness of the sample pre-treatment step was evaluated by pre-treatment of a range of glucose solutions using the **D(e)** and analysis using the **D(f)** procedure. The absorbance values (A_1_ and A_2_) were measured as per the standard procedure and an increase in absorbance between A_1_ and A_2_ was observed at higher glucose concentrations (the equivalent of >6% glucose in sample), indicating that glucose was not fully removed by the glucose oxidase/catalase pre-treatment system (Figure 9).

Low-lactose and lactose-free milk products generally contain ∼2.5% of glucose, and in such products the glucose oxidase/catalase system is capable of removing all background glucose. During the course of this single-laboratory validation (SLV) no issues were reported regarding poor removal of glucose from real samples. Where there is concern that background glucose in the samples to be analyzed is above the threshold indicated (or there is a large observed increase between A_1_ and A_2_ during the analysis i.e., A_2_–A_1_ value >0.5), it is possible to increase the time of incubation during the glucose oxidase/catalase pre-treatment procedure and a further reduction in background glucose would be expected over time.

#### (g) Selectivity.—

It is well documented that along with its primary hydrolytic function, β-galactosidase also catalyzes a transglycosylation process in which the released galactose can be transferred to lactose or pre-formed glucose, galactose, or galactooligosaccharides (GOS). This reaction is employed industrially utilizing high concentrations of lactose to produce GOS as a prebiotic ingredient. Transglycosylation also occurs to some extent during the hydrolysis of lactose for the production of low-lactose and lactose-free products. In this process, trace quantities of a range of galactosyl-glucosyl oligosaccharides are formed. While the concentrations of the various transglycosylation products are very low, they can occur at levels similar to, or greater than, that of the residual lactose present, which complicates the measurement of lactose in these samples.

**Figure 10.**
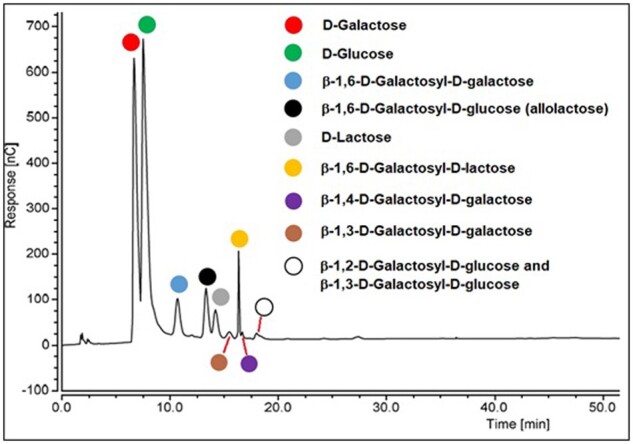
Oligosaccharide profile of a lactose-free milk product, graph obtained by HPAEC-PAD analysis.

This enzymatic method relies on the β-galactosidase mediated hydrolysis of lactose and the subsequent measurement of the glucose released as a result. The presence of the aforementioned galactosyl-glucosyl oligosaccharides in low-lactose and lactose-free samples (all of which have the potential to interfere in the measurement by falsely inflating glucose measurements if hydrolyzed) necessitates the utilization of a β-galactosidase enzyme that is selective for lactose in the presence of these potentially interfering sugars. The oligosaccharide profile of a typical commercial low-lactose milk product (obtained using HPAEC-PAD) is shown in Figure 10. A substantial number of the sugars represented (i.e., β-1,6-D-Galactosyl-galactose, β-1,3-D-Galactosyl-galactose, and β-1,4-D-Galactosyl-galactose) do not contain glucose and therefore will not inflate measured lactose values if hydrolyzed by the β-galactosidase.

**Figure 11.**
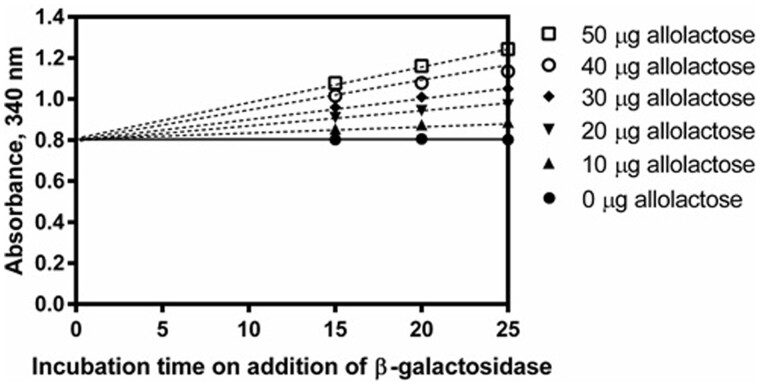
Demonstration of the effect of 0–50 μg of allolactose in the presence of 25 μg of lactose in the enzymatic determination reaction. In each mixture, linear extrapolation back to the point of addition of β-galactosidase provides the absorbance value corresponding to lactose hydrolysis only.

**Figure 12.**
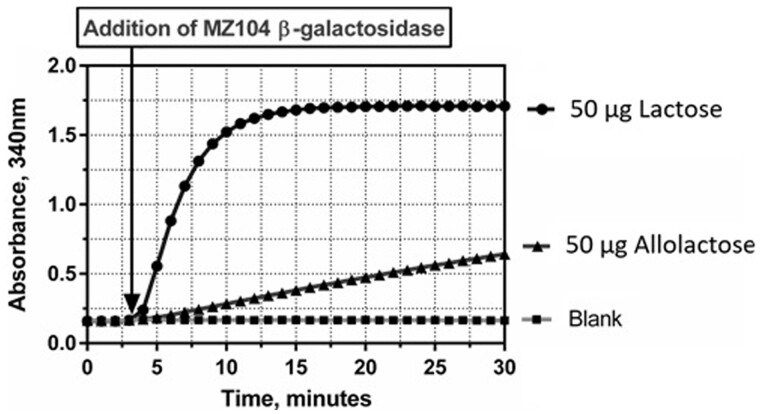
Reaction profile observed for 50 μg of lactose and 50 μg of allolactose in the enzymatic determination reaction. Note the slow, linear hydrolysis of allolactose.

**Figure 13.**
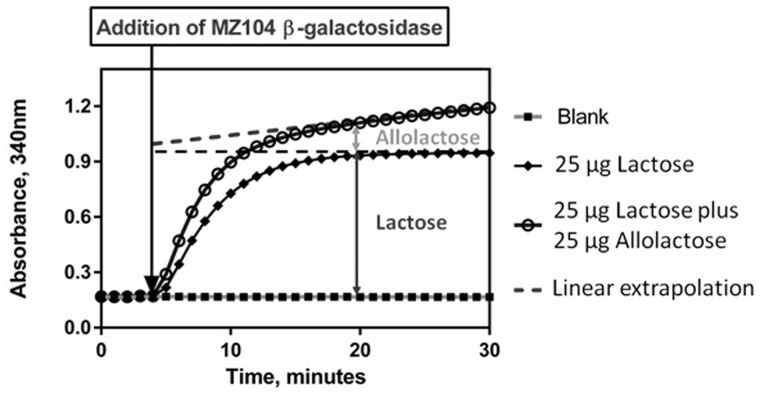
Reaction profile observed for 25 μg of lactose alongside a mixture of 25 μg of lactose and 25 μg of allolactose in the enzymatic determination reaction. Note that the slow, linear hydrolysis of allolactose in the mixture can be extrapolated back to the point of addition of β-galactosidase, which provides the absorbance value corresponding to lactose hydrolysis only.

**Figure 14.**
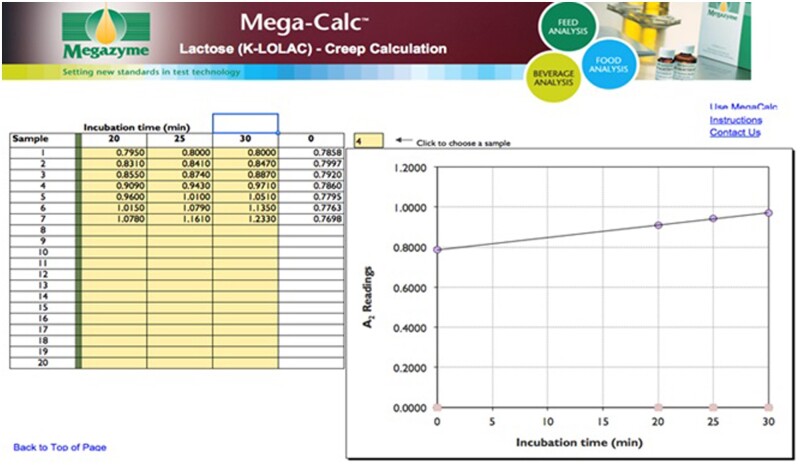
Demonstration of the application of the Megazyme creep calculator to obtain the absorbance corresponding to lactose hydrolysis in a mixture of lactose and allolactose. By entering the absorbance readings obtained at 5-min intervals after completion of the recommended 20 min β-galactosidase incubation, the absorbance value corresponding to lactose hydrolysis only is automatically generated.

β-1,6-D-Galactosyl-D-glucose (hereafter referred to as allolactose) is usually the principal component of the transglycosylation products with concentrations frequently in excess of the lactose concentration, but in certain samples it can be present in even higher quantities. Hydrolysis of allolactose in the presence of lactose was examined in a series of tests in which the lactose content was maintained at 25 μg while the allolactose content was varied from 0 to 50 μg across the linear range for lactose (Figure 11). The MZ104 β-galactosidase shows a clear preference for lactose over allolactose as the lactose hydrolysis reaction reaches completion almost immediately while the allolactose hydrolysis reaction exhibits a slow linear increase in absorbance, indicating a slow release of glucose. There is an observed linear relationship between the quantity of allolactose added to the system and the slope of the absorbance increase (referred to as “creep”). This linear relationship is expanded on in Figures 12–14. The slow hydrolysis of allolactose forms the basis for the linear extrapolation calculation.

**Figure 15.**
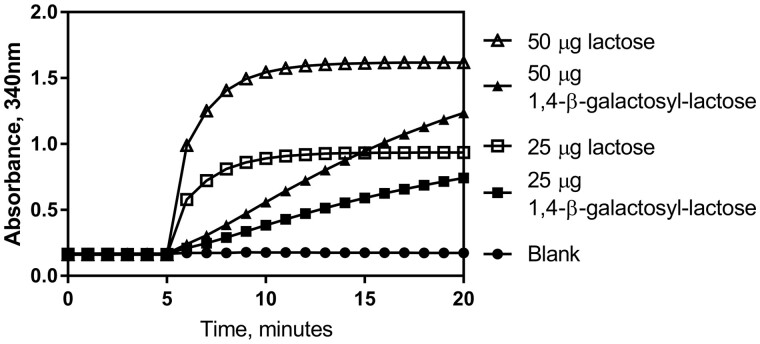
Reaction profile observed for both 25 and 50 μg of lactose and 1,4-β-D-galactosyl-lactose in the enzymatic determination reaction.

Figure 15 shows the hydrolysis profile in the enzymatic system of β-1,4-D-galactosyl-lactose when added to the test at concentrations at the middle and the top of the linear range for lactose (0, 25, and 50 µg per test). It is clear that the β-galactosidase employed can also slowly hydrolyze the β-1,4-D-galactosyl-lactose but again shows a significant preference for lactose. Any contribution to the A_3_ absorbance measurement by this sugar is also negated by the use of the linear extrapolation calculation.

The hydrolysis profiles of other minor transglycosylation products were also examined. There was no hydrolysis of β-1,6-D-galactosyl-lactose (no change in A_3_ when this sugar is present at concentrations of 0, 25, and 50 µg per test, data not shown) so there should be no inflation in results due to the presence of this sugar. There was full hydrolysis of β-1,3-D-galactosyl-glucose (data not shown), however this sugar appears in relatively low abundance in low-lactose and lactose-free products and will contribute very slightly to measured values for lactose.

#### (h) Interference.—

**Figure 16.**
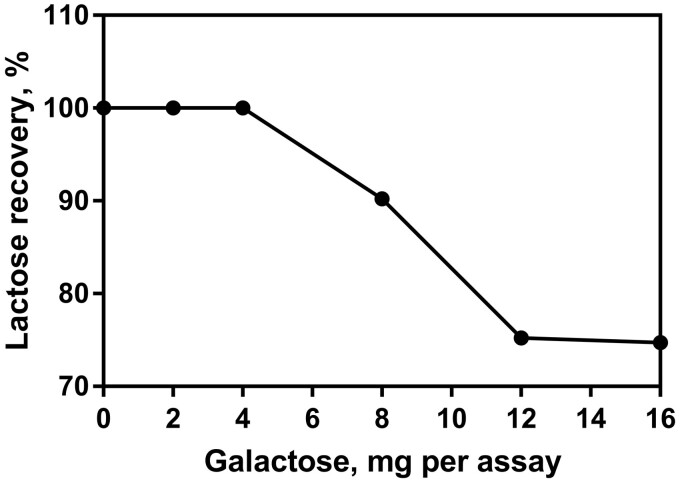
Recovery of a lactose standard when varying amounts of D-galactose are added to the enzymatic determination reaction.

Interference was first assessed during the initial stages of method development by analysis of a wide variety of “real” samples and examination of the linearity profiles of these samples. For a small percentage of samples it became clear that an increase in sample volume in the enzymatic determination reaction did not result in a linear increase in absorbance. All samples that exhibited this non-linearity were examined in greater detail and it was shown experimentally that they contained elevated levels of D-galactose (i.e., levels greater than the ∼2.5% galactose generally seen in low-lactose milks). Experiments were then carried out to examine the recovery of a known amount of lactose when an increasing amount of D-galactose is added to the system. Figure 16 shows that there was good recovery of a lactose standard up to a concentration of 4 mg D-galactose per test. Using the maximum sample volume of 0.4 mL and the **D(b)**,, this equates to >5.4 g/100 mL of D-galactose in the original sample. In order to minimize the effect of the non-linearity caused by high levels of galactose in some samples, the sample volume allowable in the enzymatic determination reaction is limited to a maximum of 0.4 mL, as all samples tested exhibited linearity up to this volume (during this investigation samples were tested over the range of 0.1–1 mL in 0.1 mL increments, data not shown).

The effect of a variety of common food components and chemicals related to food and beverage processing on the functionality of the system was investigated by the addition of these chemicals during the initial extraction steps. Known amounts of lactose and glucose were extracted as per D(c), along with 1 g of the relevant interferent. The potential interferent was added at 1 g per 50 mL extraction volume in order to simulate the addition of 10 g of sample containing 1 g of interferent [i.e., replicating a concentration of 10% w/w interferent in a real sample when using the **(D(c)**]). Lactose was included at a concentration close to the top of the linear range of the assay (the equivalent of 40 µg per test) in order to ensure that the system was close to a stress point before addition of the potential interferent. Glucose was added at a concentration close to the top of the experimentally determined range for glucose removal by the system, again ensuring that the system was close to a stress point before testing interference. It is also worth noting that the glucose concentration included in the inhibition testing was higher than the expected levels of glucose in standard low-lactose or lactose-free dairy product (typical β-galactosidase treated milk products contain ∼2.5% glucose). The results are reported in Tables 7 and 8. It is possible to conclude that there should be no interference from any chemicals listed in Table 7, the addition of which resulted in an acceptable recovery of spiked lactose and an acceptable level of glucose removal (data for glucose removal not shown).

**Table 5.** Repeatability (RSD_r_) and intermediate precision (RSD_ir_) for seven commercial food and beverage samples analyzed by two analysts over a 3-day period. Each “Extract” is a separate extraction of the sample using the relevant sample preparation example as outlined in the methods section.

Sample	M4		M5		ND4		IF1		IF3		C6		C7	
Analyst	A1^a^	A2	A1	A2	A1	A2	A1	A2	A1	A2	A1	A2	A1	A2
Day 1, Extract 1	2.536	2.255	67.624	66.268	2.491	2.654	2.784	2.885	2.255	2.203	150.012	146.015	32.651	31.062
Day 1, Extract 1	2.566	2.361	68.625	69.148	2.492	2.741	2.953	2.752	2.221	1.994	147.142	147.117	32.767	30.981
Day 1, Extract 2	2.731	2.387	63.149	69.137	2.962	2.531	2.907	2.806	2.36	2.025	142.023	123.987	30.929	31.388
Day 1, Extract 2	2.611	2.421	60.746	67.688	2.998	2.425	2.821	2.811	2.476	2.137	141.574	121.901	31.01	31.394
Day 2, Extract 1	2.293	1.974	59.332	66.117	2.531	2.827	2.722	2.843	2.079	2.217	137.985	124.874	31.301	31.458
Day 2, Extract 1	2.051	2.064	66.064	66.262	2.493	2.771	2.779	2.668	2.165	2.104	138.994	124.888	31.219	31.446
Day 2, Extract 2	2.393	1.954	66.262	66.245	2.559	2.418	2.859	2.838	2.13	2.319	141.125	121.925	31.027	32.261
Day 2, Extract 2	2.565	1.93	67.554	69.643	2.518	2.486	2.928	2.784	2.015	2.04	138.367	125.754	30.107	32.57
Day 3, Extract 1	2.459	2.331	66.157	69.404	2.473	2.293	2.859	2.827	2.373	2.102	141.221	129.777	30.457	31.749
Day 3, Extract 1	2.462	2.284	67.182	69.501	2.684	2.29	2.603	2.812	2.108	2.13	146.012	127.801	30.859	32.82
n	10	10	10	10	10	10	10	10	10	10	10	10	10	10
Mean	2.467	2.196	65.270	67.941	2.620	2.544	2.822	2.803	2.218	2.127	142.446	129.404	31.233	31.713
STDEV^b^	0.190	0.194	3.132	1.572	0.199	0.195	0.105	0.059	0.147	0.099	4.009	9.358	0.854	0.629
RSDr	7.686	8.848	4.799	2.314	7.599	7.682	3.729	2.110	6.641	4.640	2.814	7.232	2.734	1.985
RSDir	8.233		3.531		7.640		2.922		5.661		4.917		2.357	

a A = Analyst. ^b^STDEV = Standard deviation.

**Table 6.** Recovery data for 36 food and beverage samples spiked with a known amount of lactose

Sample identifier	Measured lactose (sample), mg/100 mL or mg/100g^a^	Expected lactose (spike), mg/100 mL or mg/100g^b^	Measured lactose (spike), mg/100 mL or mg/100g^c^	Recovery (spike), %
M1	4.44	10	10.19	101.90
M2	26.61	10	9.74	97.40
M3	<0.89	10	12.49	110.60
M4	2.33	10	10.24	102.40
M5	66.61	10	9.98	99.80
Y1	27.5	10	10.14	101.4
Y2	15.1	10	9.85	98.5
Y3	38.8	10	10.15	101.5
F1	2.54	7	7.82	111.65
F2	<1.48	7	7.10	101.47
F3	<1.48	7	7.92	113.21
F4	2.65	7	7.27	103.91
F5	<1.48	7	7.39	105.58
F6	28.14	10	10.04	100.41
F7	<1.48	10	10.12	101.24
C1	<1.48	10	10.66	106.60
C2	2.35	10	9.99	99.90
C3	1.58	10	11.41	114.10
C4	<1.48	10	11.15	111.50
C5	<1.48	10	9.77	97.71
C6	135	10	10.11	101.11
C7	31.54	10	11.01	110.14
IF 1	2.81	5	5.12	102.40
IF 2	4.41	5	5.15	103.13
IF 3	2.17	5	5.34	106.26
IF 4	<0.89	5	5.18	103.14
IF 5	<0.89	5	4.99	99.16
IF 6	<0.89	5	4.66	93.21
IF 7	1.28	5	5.03	100.61
IF 8	2.42	10	10.85	108.50
IF 9	3.23	10	9.86	98.64
IF 10	<0.89	10	10.93	109.34
IF 11	<0.89	10	10.30	103.02
ND1	<0.89	10	11.28	112.82
ND2	<0.89	10	10.68	106.84
ND3	73.51	10	10.75	107.51
ND4	2.58	10	10.21	102.11
CRM1	9.89	10	10.16	101.61
CRM2	222.70	100	100.88	100.88
CRM3	16.56	10	10.78	107.85
CRM4	4924.13	1000	944.47	94.44
CRM5	23953.68	5000	5027.06	100.54
CRM6	21398.12	5000	5351.96	107.04
CRM7	22872.30	5000	5414.21	108.28
CRM8	2638.40	1000	945.52	94.52
CRM9	3794.54	1000	989.04	98.90
CRM10	8737.54	5000	4748.97	94.97

a Measured lactose (sample)—Concentration of lactose measured in sample without spiking.

b Expected lactose (spike)—Concentration of spike added to test.

c Measured lactose (spike)—Concentration of spike measured (taking away separately determined value for sample).

**Table 7.** Percentage recovery of a lactose standard when extracted as per **D(c)** with 1 g of the specified potential interferent in the extraction (the equivalent of 1 g per 10 g of sample, or 10% w/w interferent). Samples were treated as per **D(e)** and then analyzed in the “*enzymatic determination reaction.—All samples*” using the maximum sample volume of 0.4 mL per test

Chemical	CAS number	Interferent, % (equivalent g per 100 g of sample weight)	Lactose recovery, %
Salts			
Calcium chloride	10043-52-4	10	98.51
Magnesium chloride	7786-30-3	10	99.53
Manganese chloride	7773-01-5	10	97.52
Potassium chloride	7447-40-7	10	100.41
Sodium chloride	7647-14-5	10	100.49
Zinc chloride	7646-85-7	10	99.246
Sugar alcohols			
Erythritol	149-32-6	10	98.88
Glycerol	56-81-5	10	99.53
Lactitol	585-86-4	10	97.84
Maltitol	585-88-6	10	98.31
Mannitol	87-78-5	10	99.87
*myo*-Inositol	87-89-8	10	100.91
Sorbitol	50-70-4	10	99.38
Xylitol	87-99-0	10	97.49
Organic acids			
Acetic acid	64-19-7	10	99.64
Ascorbic acid	50-81-7	10	93.61
Aspartic acid	617-45-8	10	98.59
Citric acid	77-92-9	10	99.21
Galacturonic acid	685-73-4	10	101.95
Gluconic acid	133-42-6	10	98.66
Glucuronic acid	6556-12-3	10	99.22
Glutamic acid	617-65-2	10	100.70
Lactic acid	50-21-5	10	99.81
Malic acid	6915-15-7	10	98.04
Succinic acid	110-15-6	10	97.97
Tartaric acid	526-83-0	10	100.92
Amino acids			
Glycine	56-40-6	10	104.41
Histidine	71-00-1	10	104.63
Isoleucine	73-32-5	10	99.34
Lysine	56-87-1	10	97.27
Tryptophan	54-12-6	10	100.81
Tyrosine	60-18-4	10	97.80
Valine	516-06-3	10	97.40
Other			
Aspartame	22839-47-0	10	99.80
Casein	9000-71-9	10	101.12
Cellobiose	528-50-7	10	104.649
Cellotriose	33404-34-1	10	102.05
Fructan	9013-95-0	10	99.93
Galactomannan	900-30-0	10	101.94
Glycerol triacetate	102-76-1	10	99.84
Isomalt	64519-82-0	10	98.33
Lactulose	4618-18-2	10	101.14
Maltose	69-79-4	10	98.567
Maltotriose	1109-28-0	10	101.37
Sodium alginate	9005-38-3	10	97.73
Starch	9005-25-8	10	102.89
Sucralose	56038-13-2	10	97.35
Sucrose	57-50-1	10	100.5
β-glucan	9012-72-0	10	101.14

Where interference was observed, either by a reduced A_3_ value (poor recovery of lactose) or an inflated A_2_ value (poor removal of glucose), the test was repeated over a range of inhibitor concentrations until both a full recovery of lactose and full removal of glucose were achieved. These results are shown in Table 8. Elevated levels of acetaldehyde, arginine, and copper(I) sulphate do cause inhibition in the enzymatic determination reaction (reduced A_3_ value resulting in a poor recovery of lactose standard) and not in the glucose oxidase/catalase system (glucose was removed successfully), suggesting that these chemicals have an inhibitory effect on either the glucose determination enzymes (HK/G6PDH/6PGDH) or the β-galactosidase enzyme utilized in the system. In practice, it is highly unlikely that acetaldehyde (or other aldehydes), arginine, or copper(I) sulphate will be present in food or beverage samples at levels anywhere near the experimentally determined levels shown to cause interference.

**Table 8.** Table showing percentage recovery of a lactose standard when extracted as per **D(c)** with varying amounts of the specified interferent in the extraction (the equivalent of 0.2–0.8 g per 10 g of sample, or 2–8% w/w interferent). Extracted samples were treated as per **D(e)** and then analyzed in the Enzymatic determination reaction.—All samples procedure using the maximum sample volume of 0.4 mL per test

Chemical	CAS number	Interferent, % (equivalent g per 100 g of sample weight)	Lactose recovery, %
Acetaldehyde	75-07-0	2	100.01
		4	98.123
		6	95.496
		8	93.830
Arginine	7200-25-1	2	98.864
		4	98.093
		6	94.956
		8	92.411
Copper (I) sulphate	17599-81-4	2	99.543
		4	95.581
		6	96.721
		8	93.656

The possibility that there may be some interference due to the presence of residual β-galactosidase (remaining after the industrial removal or reduction of lactose) was also taken into consideration. In this case the concern exists that any residual β-galactosidase present in the sample could theoretically further hydrolyze any lactose present in the sample upon extraction (resulting in an under-reporting of lactose in the sample). The treatment of samples using the **D(b)** removes the potential for interference as the immediate treatment with Carrez reagents fully denatures and deactivates any protein present. In samples such as cheeses and other solid samples where solubilization is required before analysis, in theory it is possible that β-galactosidase could be active. In practice, however, no such interference was observed during the numerous recovery experiments using real solid samples (Table 6).

It is also possible to further use the “real” sample recovery data as an indicator of potential inhibition or interference. During recovery experiments a wide range of samples were tested for lactose (results shown in Table 6). Where a good recovery of lactose was achieved it is possible to say that no sample components inhibited the measurement system, further proving that the method does not suffer from any major drawbacks in terms of chemical inhibition.

Where there is concern that there may be interfering substances present in the sample for analysis, an internal standard can be included by the analyst. Quantitative recovery of this standard would be expected. Losses in sample handling and extraction are identified by performing recovery experiments (i.e., by adding lactose to the sample in the initial extraction steps).

#### (i) Stability.—

**Figure 17.**
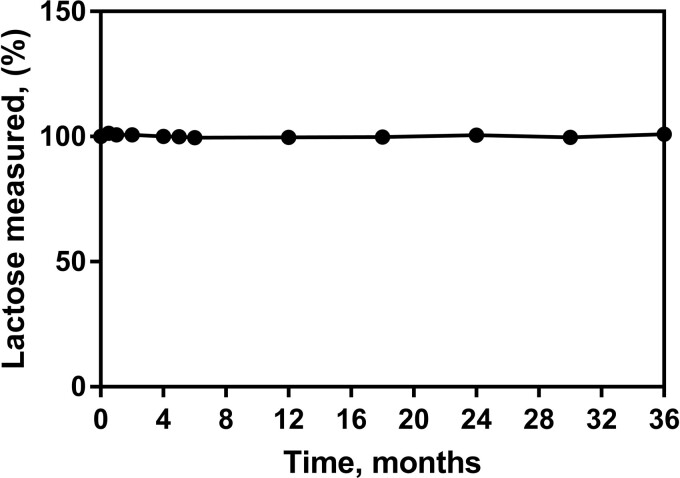
Stability of one set of kit components over a period of 36 months. The lactose standard was assayed at the highest concentration (50 μg per cuvette) in order to thoroughly test the system for stability. Note that glucose removal was also tested at these times and no issues were reported (data not shown).

The Lactose Assay Kit is formulated by Megazyme with a two-year stability guarantee when the components are stored as described on the individual kit component label. Kit components may be provided with longer stability guarantees, the user can find this information on the product label (specific expiry date is stated on each component). Regular quality control testing is performed in the Megazyme QC laboratory. The functionality of one set of kit components over a period of 3 years can be seen in Figure 17. As the Lactose Assay Kit has been commercially available for a number of years, the historical data available from the QC laboratory shows that the stability of each component does not vary from batch to batch (data not shown).

**Figure 18.**
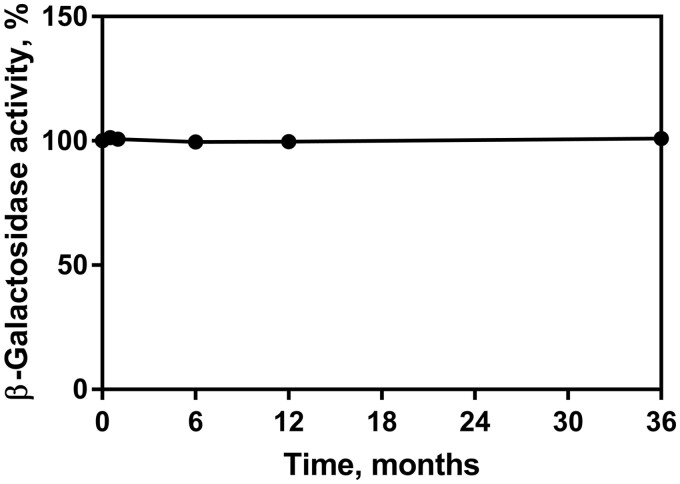
Stability of β-galactosidase (Suspension 6) over a period of 36 months. The enzyme was stored at 4°C (recommended storage) and assayed for activity (U/mL) which was then plotted as percentage activity remaining.

The nature of the enzyme kit components (Suspension 5, HK/G6PDH/6PGDH and Suspension 6, β-galactosidase) is such that they are the most likely to display poor stability. The HK/G6PDH/6PGDH has been used for decades in this formulation in several other Megazyme test kits and has not exhibited any stability issues over time (data not shown). A detailed stability study was carried out on the β-galactosidase suspension, measuring enzyme activity. Enzyme storage stability at 4°C (recommended storage for β-galactosidase) is shown in Figure 18 (U/mL values are converted to % activity). Note that a significant allowance has been made when formulating the enzyme components for any potential loss of activity that may occur (due to incorrect storage conditions or similar) as both enzyme suspensions are supplied greatly in excess of the U/mL concentration required for full functionality in the test.

## Discussion

This SLV included investigation into a variety of performance characteristics including working range, LOD, LOQ, trueness (*bias*), precision (repeatability and intermediate precision), selectivity, interference, robustness, and stability.

The assay was shown to be linear over a range of 1–25 µg per test for glucose and 1–50 µg per test for lactose. For samples treated as per the method outlined for liquid samples the working range of 1–50 µg of lactose per cuvette equates to a concentration range of 1.35–67.5 mg/100 mL of lactose in the original sample when using the maximum assay volume of 0.4 mL. For samples treated as per the method outlined for solid samples the working range of 1–50 µg of lactose per cuvette equates to a concentration range of 2.25–112.5 mg/100g of lactose in the original sample when using the maximum assay volume of 0.4 mL.

The LOD and LOQ were determined for the enzymatic determination reaction and subsequently for both liquid and solid samples, when sample are analyzed using the relevant sample preparation example as outlined in the methods section.

Trueness was tested using in-house lactose reference materials and the results were excellent across a range of concentrations. Experiments showed good correlation between results achieved using this procedure and expected results for 9 out of 10 harmonization materials tested (using values stated by the manufacturer, obtained by HPLC). Good correlation was not achieved with the sodium caseinate material, but this difference was shown to not be statistically significant when taking into account the standard deviation of the reference method.

A set of seven commercial samples were analyzed to provide repeatability and intermediate precision data. Samples were extracted and analyzed by two analysts over a 3-day period. Each sample was extracted twice per day by each analyst and analyzed in duplicate. For these samples (including two lactose-free milk samples, two infant formula samples, two cheese samples, and an adult nutritional drink), the highest RSD_r_ value was 8.9, while the highest RSD_ir_ value was 8.3.

A set of 36 samples (covering a wide range of matrix types) were analyzed and the recovery of a spiked lactose standard was measured. For infant formula samples, samples were spiked with a lactose standard at 5 mg/100 mL (for samples referred to as “lactose-free” by the manufacturer) and 10 mg/100 mL (for samples referred to as “low-lactose” by the manufacturer). Recoveries across the 11 infant formula samples varied from 93.2–109.34%. For all other samples tested within the lower range (10–100 mg/100 g), recoveries varied from 93.21–114.10%. A number of the Muva Kempten reference materials contained lactose at concentrations higher than the 1000 mg/100 g level that could be considered “low-lactose”. These samples were included in the SLV in order to demonstrate that traditional dairy samples can be analyzed using this method. Recoveries obtained for samples in the higher range (i.e., >100 mg/100g or mL) varied from 94.44–108.28%.

Robustness testing included the examination of incubation temperature in the enzymatic determination reaction (20, 25, and 37°C), time at which absorbance measurement is taken (10, 12.5, 15, 17.5, 22.5, 25, and 27.5 min), units of β-galactosidase per test (2.75–44 U per test), and glucose removal in the **D(e)** step. No parameter investigated during robustness testing was found to influence the result in any way when the test is performed as described within the methods section and for the sample types outlined.

The method can be considered to be selective for lactose in the matrices specified, under the assumption that the user utilizes the linear extrapolation calculation effectively where interfering sugars are present (as indicated by the presence of a “creep” i.e., a gradual increase in absorbance values over time after the expected assay completion time). Minor overestimation of lactose is observed where samples contain β-1,3-galactosyl-glucose, however this oligosaccharide is present in relatively low concentrations in low-lactose and lactose-free products.

All assay components were shown to have at least 3 years stability when stored as recommended and both enzyme components (HK/G6PDH/6PGDH and β-galactosidase) exhibit excellent stability for at least 3 years when stored as recommended (4°C).

This SLV shows that the Lactose Assay Kit (K-LOLAC) is fit for purpose and applicable for the determination of lactose in low-lactose or lactose-free products, including infant formula and adult nutritional drinks, conventional dairy samples, and a variety of food samples.

The test is user-friendly and the assay, excluding pre-treatment, is complete within 30 min.

## Conclusion

It is possible to conclude that the Lactose Assay (K-LOLAC) is fit-for-purpose. The assay meets all requirements set out in AOAC SMPR 2018.009.

## Conflict of Interest

The authors report the following details of affilitation or involvement in an organisation with a financial interest in the subject matter or materials discussed in this manuscript: for the duration of the research presented the authors were employed by Megazyme, manufacturer of the K-LOLAC test kit. 
